# Tumor immune microenvironment changes are associated with response to neoadjuvant chemotherapy and long-term survival benefits in advanced epithelial ovarian cancer: A pilot study

**DOI:** 10.3389/fimmu.2023.1022942

**Published:** 2023-03-13

**Authors:** Guangming Cao, Dingchao Hua, Jinfeng Li, Xuefang Zhang, Zhiqiang Zhang, Bei Zhang, Ting Bei, Lina Cui, Shiqing Chen, Shuzhen Wang, Lei Zhu

**Affiliations:** ^1^ Department of Obstetrics and Gynecology, Beijing Chao-Yang Hospital, Capital Medical University, Beijing, China; ^2^ Department of Medical Affairs, 3D Medicines Inc., Shanghai, China

**Keywords:** epithelial ovarian cancer, neoadjuvant chemotherapy, tumor immune microenvironment, treatment efficacy, prognosis

## Abstract

Little is known about the association between efficacy of neoadjuvant chemotherapy (NACT)/survival and the dynamic change of tumor immune environment (TIME) during treatment in epithelial ovarian cancer (EOC). This study investigated the TIME landscape of treatment-naive EOC tumors using multiplex immunofluorescence and associated the TIME before and after platinum-based NACT with treatment efficacy and prognosis in 33 patients with advanced EOC. NACT significantly increased the density of CD8^+^ T cells (*P* = 0.033), CD20^+^ B cells (*P* = 0.023), CD56 NK cells (*P* = 0.041), PD-1^+^ cells (*P* = 0.042), and PD-L1^+^CD68^+^ macrophages (*P* = 0.005) in the tissue specimens. Response to NACT was evaluated using CA125 response and chemotherapy response score (CRS). Compared with the non-responders, the responders displayed a larger proportion of tumors showing increase in the infiltration of CD20^+^ cells (*P* = 0.046) and in the M1/M2 ratio (*P* = 0.038) as well as fewer tumors showing increase in the infiltration of CD56^bright^ cells (*P* = 0.041). No association was found between pre-NACT TIME and response to NACT. Density of pre-NACT CD8^+^ cells was positively associated with longer progression-free survival (PFS) (*P* = 0.011) and overall survival (OS) (*P* = 0.048). Post-NACT CD20^+^ and CD163^+^ macrophages (M2) infiltrates were associated with prolonged (*P* = 0.005) and shortened PFS (*P* = 0.021), respectively. Increase in the density of CD4^+^ T cells was predictive for longer PFS (*P* = 0.022) and OS (*P* = 0.023). In the multivariate analysis, high density of CD8^+^ cells pre-NACT (*P* = 0.042) were independently associated with improved OS.

## Introduction

Ovarian cancer is the most lethal gynecologic malignancy (1). Epithelial ovarian cancer (EOC) accounts for about 90% of ovarian malignancies (2), and the majority of EOC patients present with advanced tumor stage at diagnosis. The prognosis of patients with a late-stage disease is dismal, with a 5-year survival rate of less than 30% (3). Complete resection combined with platinum-based chemotherapy has been the primary choice for patients with EOC. In advanced and metastatic EOC where complete resection is not feasible, neoadjuvant chemotherapy (NACT) followed by interval debulking surgery (IDS) and adjuvant chemotherapy is an alternative (4, 5). However, NACT is associated with a moderate survival benefit, and with NACT, a considerable proportion of patients with advanced disease still succumb to recurrence (4, 6–8).

Increasing clinical evidence has suggested that analyses of tumor immune environment (TIME) of treatment-naïve tumor has allowed for identifying components of immune contexture that are beneficial [including tumor-infiltrating lymphocytes (TILs): CD8^+^ cytotoxic cells, and CD3^+^ T cells] or deleterious (including Foxp3^+^ T cells, and CD163^+^ macrophages) to ovarian cancer patients (9–12). Other studies have attempted to unveil the effect of conventional cytotoxics on TIME (13–15). Little, however, is known about the association of TIME changes upon NACT with NACT efficacy and survival benefit. Findings from that analyses may identify predictive signatures for treatment efficacy and survival as well as uncover potential pathways, mechanisms, and biomolecules that could be co-targeted in new treatment combinations to prolong disease control in advanced EOC.

This study aimed to investigate the association of TIME orientation with NACT efficacy and survival outcomes in a cohort of annotated EOC tissue biopsies obtained at diagnosis and at IDS following chemotherapy.

## Method

### Study design

We retrospectively reviewed EOC patients who received at least one cycle of platinum-based NACT between February 22, 2011, and November 15, 2018, at Beijing Chao-Yang Hospital. Patients who had III/IV EOC as defined by the International Federation of Gynecology and Obstetrics (FIGO) and pre-NACT and post-NACT tumor tissue specimens were eligible. Pre-NACT samples were biopsy specimens obtained with diagnostic laparoscopy, and post-NACT samples were taken from the tumor tissues obtained at IDS. All the samples were subjected to mIF to evaluate TIME. Depending on data availability and sample quality, the association of TIME with NACT efficacy and survival outcomes was explored. The flow diagram of the study design was shown in **Supplementary Figure S1**. This study was approved by the ethics committee of Beijing Chao-Yang Hospital (2021-Science-639).

### Treatment

All patients received NACT of taxane/platinum combinations for a median of three cycles (range, 2–4 cycles) before IDS. No other treatment such as radiation or endocrine therapy was performed before IDS. For IDS, all patients underwent surgery with the intent to achieve complete cytoreduction with no gross residual tumor. Subsequently, at the discretion of the physician, patients underwent 4−8 cycles of adjuvant chemotherapy.

### Evaluation of response to NACT

As radiological evaluation of response using response evaluation criteria in solid tumors (RECIST) is not suitable for EOC characterized of cancer dissemination, we used CA125 response and CRS, which are extensively utilized in clinical practice, to evaluate the response to platinum-based NACT. CA125 response using gynecological cancer intergroup (GCIG) CA125 criteria (16) and chemotherapy response score (CRS) recommended by the European Society for Medical Oncology and European Society for Gynecological Oncology (ESMO-ESGO) (17). CA125 response is defined as at least a 50% reduction in CA125 levels from a pretreatment sample. The response must be confirmed and maintained for at least 28 days. Patients can be evaluated according to CA125 only if they have a pretreatment sample that was at least twice the upper limit of the reference range within two weeks before starting the treatment. CRS is a three-tier system based on pathological reaction of surgical specimens in which CRS1 shows no or minimal tumor response, CRS2 shows moderate/appreciable tumor response, and CRS3 usually shows complete or near-complete response. In this study, patients with samples scored as CRS3 or CRS2 were defined as CRS responders and patients with biopsies scored as CRS1 were non-responders.

### Tumor microenvironment by multiplex immunofluorescence

Surgical tissue specimens were subjected to the examination of the TIME, which was performed as previously described by 3D Medicines, Inc., a College of American Pathologists (CAP)-accredited and Clinical Laboratory Improvement Amendments (CLIA)-certified laboratory (18). Primary antibodies targeting CD163, CD68, PD-1, PD-L1, CD3, CD4, CD8, CD56, CD20, Foxp3 and pan-CK or S100 were sequentially applied to FFPE tissue slides (**Supplementary Table S1**). After the incubation with secondary antibodies, corresponding reactive Opal fluorophores, and nuclei acids staining reagent DAPI, multiplex stained slides were scanned using a Vectra Polaris Quantitative Pathology Imaging System (Akoya Biosciences), which was configured to capture fluorescent spectra at 20 nm wavelength intervals from 440 nm to 780 nm with a fixed exposure time. All scans for each slide were then superimposed to obtain a single image. Unstained and monoplex stained slide images were applied to extract Tissue autofluorescence was extracted with unstained and monoplex stained slide images. Fluorescence images were analyzed using the APTIME software developed by 3D Medicines. Tumor parenchyma and stroma were differentiated according to CK staining. The CK-positive area with DAPI staining was defined as tumor region, and the CK negative area with DAPI staining was considered stroma region. The quantities of various cell subsets were expressed as the count number of positively stained cells per square millimeter (cells per mm^2^). The density of immune cell subsets in tumor and stroma regions were figured out by detecting signal channel or multiple-channel, namely CD3^+^, CD3^+^CD4^+^, CD8^+^, Foxp3^+^, PD-1^+^CD8^+^, CD4^+^Foxp3^+^ (Treg), CD68^+^CD163^-^ (M1 macrophage), CD68^+^CD163^+^ (M2 macrophage), PD-L1^+^ CD68^+^, CD56 bright (NK cell), CD56 dim (NK cell), etc. The co-occurrence of CD3^+^ T cells and CD20^+^ B cells indicates the formation of tertiary lymphoid structures (TLS).

### Statistical analysis

For paired comparisons we used wilcoxon matched-paired signed-ranked test, and for non-paired comparisons, wilcoxon rank-sum test was considered. Survival curve and median survival time were depicted using Kaplan-Meier survival curve. Nonparametric log-rank testing was employeed to determine the differences among different immune cell infiltration groups, with a median cutoff used to distinguish low vs high levels. The hazard ratio (HR) and 95% confidence interval (CI) were estimated using the Cox proportional hazard model. Univariate survival analyses were performed using the Cox proportional-hazards model, and multivariate Cox regression analysis was performed to select independent prognostic factors. A two-side *P* ≤ 0.05 was considered to represent a statistically significant difference. Statistical analysis was performed using R software (version 3.6.1).

## Results

### Clinicopathological characteristics of patients

A total of 33 EOC patients received NACT + IDS and had paired pre-NACT and post-NACT tumor specimens. The median age of the cohort was 60 years [interquartile range (IQR), 53−65]. Serous carcinoma was the predominant histologic subtype (26/33, 78.8%), and most of the patients had FIGO stage III disease (30/33, 90.9%). Almost all the tumors were grade 3 (32/33, 97.0%). Approximately half of the patients had lymph node metastasis. The median CA125 level was 1632.0 U/mL at baseline. Seventy-six percent of patients received two cycles of NACT (2 cycles, n = 25; 3 cycles, n = 5; 4 cycles, n = 3). CA125 response to 1^st^ cycle NACT was assessed in 31 cases (CA-125 1^st^ cycle responder, n = 22; CA-125 1^st^ cycle non-responder, n = 9), and chemotherapy response score was evaluated in 25 cases (CRS1, n = 8; CRS2, n = 16; CRS3, n = 1). Twenty-six (26/33, 78.8%) patients were platinum-sensitive, and the rest seven cases were platinum-resistant. The median follow-up of the cohort was 40.9 months. Complete cytoreduction was achieved at interval surgery in 19 of the 33 patients (57.6%) (**Table 1**).

**Table 1:** Clinicopathological characteristics of patients (n=33).

Variable	n (%) N = 33
**Age at diagnosis, Median (IQR)**	60 (53, 65)
Histologic type	
Serous	26 (78.8%)
Clear cell	3 (9.1%)
Mucinous	1 (3.0%)
Mixed	3 (9.1%)
2014 FIGO stage	
III	30 (90.9%)
IV	3 (9.1%)
Histologic tumor grade	
Grade 1	1 (3.0%)
Grade 3	32 (97%)
ECOG	
1	7 (21.2%)
2	20 (60.6%)
3	6 (18.2%)
**Baseline CA125 (U/mL),Median (IQR)**	1632 (91-17980)
Lymph node metastasis	
No	18 (56.3%)
Yes	14 (43.7%)
Unknown	1
**NACT cycles, Median (Min, Max)**	2 (2, 4)
**Adjuvant chemotherapy cycles, Median (Min, Max)**	6 (4, 8)
CA125 1st cycle response	
No	9 (29.0%)
Yes	22 (71.0%)
Unknown	2
Chemotherapy response score	
1	8 (32.0%)
2	16 (64.0%)
3	1 (4.0%)
R0	
Yes	19 (57.6%)
No	14 (42.4%)
Platinum sensitivity	
Platinum sensitive	26 (78.8%)
Platinum resistant	7 (21.2%)

FIGO, International Federation of Gynecology and Obstetrics; ECOG, Eastern Cooperative Oncology Group score; NACT, neoadjuvant chemotherapy; IQR, interquartile range.

### TIME changes upon NACT

**Figure 1:**
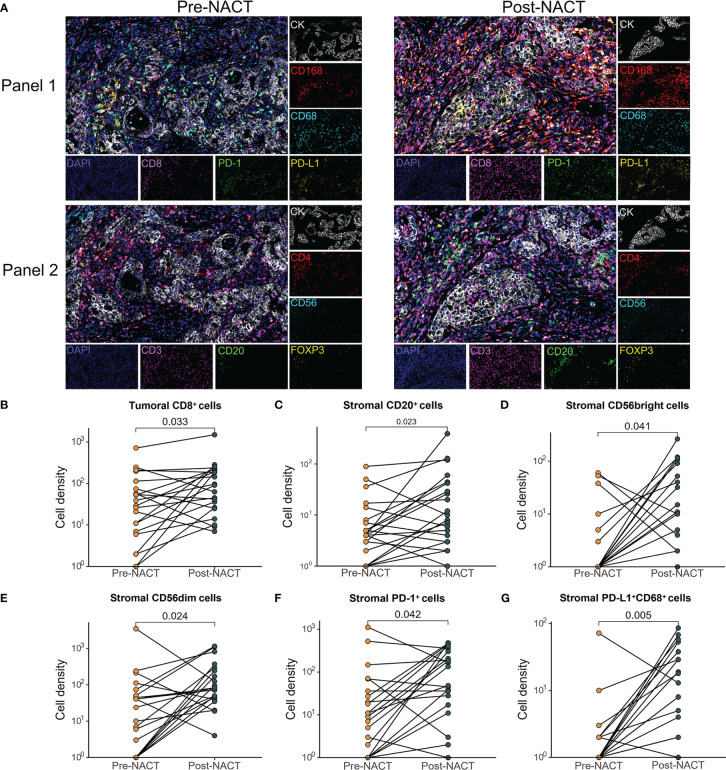
Comparison of changes in the tumor immune microenvironment between pre- and post-NACT matched tumor samples (n=21). **(A)** Images of representative mIF results. Pre-NACT mIF image on left, post-NACT mIF image on right. **(B–G)** Comparison of density of **(B)** tumoral CD8^+^ cell; **(C)** stromal CD20^+^ cell; **(D)** stromal CD56bright cell; **(E)** stromal CD56dim cell; **(F)** stromal PD-1^+^ cell; **(G)** stromal PD-L1^+^ CD68^+^ cells between pre- and post-NACT tumor samples. P value, Wilcoxon matched-paired signed-ranked test for paired samples.

Using mIF, the tissue biopsies were stained for multiple markers of immune cells. The baseline cell density in the malignant cell areas and adjacent stromal areas of 30 patients was shown in **Supplementary Figure S2**. Depending on sample quality, we analyzed the infiltration of tumor immune cell subsets in paired tumor tissue specimens from 21 patients to examine the TIME changes of EOC with NACT. Upon NACT, in the tumor center the density of CD8^+^ T cells, significantly increased (pre-NACT vs. post-NACT: CD8^+^ T cell, *P* = 0.033) (**Figures 1A, B**). The density of CD68^+^CD163^+^ M2 macrophages significantly decreased (pre-NACT vs. post-NACT: CD68^+^CD163^+^ T cell, *P* = 0.039) (**Supplementary Figure S3**). In the tumor stroma, significantly increased infiltration of CD20^+^ B cells, CD56 NK cells, PD-1^+^ cells and PD-L1^+^CD68^+^ macrophages was observed (pre-NACT vs. post-NACT: CD20^+^ B cell, *P* = 0.023; CD56^bright^ cells, *P* = 0.041; CD56^dim^ cells, *P* = 0.024; PD-1^+^ cells, *P* = 0.042; PD-L1^+^CD68^+^ macrophages, *P* = 0.005) (**Figures 1A–G**). The expression of PD-L1 in the post-NACT tumor specimens was significantly elevated than in the pre-NACT tumor tissues (*P* < 0.001, Data not shown). No significant difference was found in the density of other immune cell subsets, including CD3^+^, CD4^+^Foxp3^+^, CD4^+^, CD68^+^CD163^-^ and PD-1^+^CD8^+^ (**Supplementary Table S2; Supplementary Figure S3**) between pre-NACT and post-NACT tissue biopsies.

### Association of NACT efficacy with TIME

**Figure 2:**
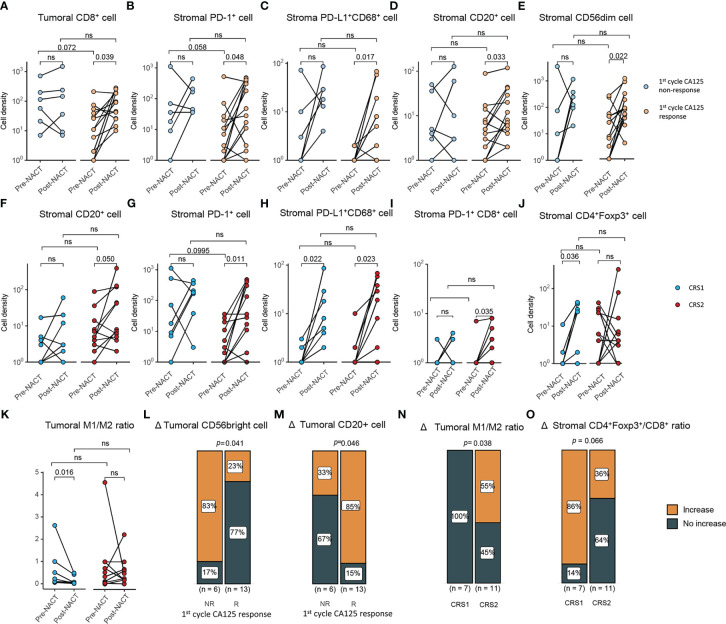
Correlations between the change of tumor immune microenvironment and response to NACT. **(A–D)** Comparison of density of **(A)** tumoral CD8^+^ cells, **(B)** stromal PD-1^+^ cells, **(C)** stromal PD-L1^+^ CD68^+^ cell, **(D)** stromal CD20^+^ cell, **(E)** CD56dim cells between pre- and post-NACT tumor samples in CA125 responders and CA125 non-responders (CA125 responders, n=13; CA125 non-responders, n=6). **(F–J)** Comparison of density of **(F)** stromal CD20^+^ cell **(G)** stromal PD-1^+^ cells, **(H)** stromal PD-L1^+^ CD68^+^ cell, **(I)** stromal PD-1^+^CD8^+^ cell, **(J)** stromal CD4^+^Foxp3^+^ cells between pre- and post-NACT tumor samples in CRS1 and CRS2 group (CRS1, n=7; CRS2, n=11). **(K)** Tumoral M1/M2 macrophage density ratio of pre- and post-NACT tumor samples. **(L, M)** Density change in CD56 bright cells **(L)** and CD20+ cells **(M)** between the CA125 responders and non-responders after the first cycle of NACT treatment. **(N, O)** Density change in CD56 bright cells **(N)** and CD20+ cells **(O)** between the CRS1 and CRS2 groups. P value, Wilcoxon matched-paired signed-ranked test for paired samples. ns, no significance.

Response to platinum-based chemotherapy was evaluated using CA125 response and CRS. We examined the association of NACT response with the change of TIME during NACT and TIME pre- and post-NACT. The 1^st^ cycle CA-125 changes were determined in 19 of 21 patients with TIME information of both pre- and post-NACT tissue specimens. Compared with pre-NACT specimens, there was a significant increase in the density of CD8^+^ T cells in the tumor areas of post-NACT tissue biopsies in the 1^st^ cycle CA125 responders (*P* = 0.039). We also observed a significant increase in the infiltrates of PD-1^+^ cells (*P* = 0.048), PD-L1^+^CD68^+^ macrophages (*P* = 0.017), CD20^+^ B cells (*P* = 0.033), and CD56^dim^ (*P* = 0.022) (**Figures 2A–E**) in the stroma of the 1^st^ cycle CA125 responders. In the 1^st^ cycle CA125 non-responders, the density of CD56^bright^ NK cells tended to increase after NACT (*P* = 0.059, **Supplementary Tables S3–S6**).

CRS was assessed in 18 patients, of whom 7 were CRS1 and 11 were CRS2. In CRS2 biopsies, NACT significantly increased the infiltration CD20^+^ B cells (*P* = 0.050), PD-1^+^ cells (*P* = 0.011), PD-L1^+^CD68^+^ macrophages (*P* = 0.023) and PD-1^+^CD8^+^ cells (*P* = 0.035) in the stoma of CRS2 responders (**Figures 2F–I**). NACT significantly increased infiltrates of CD4^+^Foxp3^+^ Treg cells (*P* = 0.036) and PD-L1^+^CD68^+^ macrophages (*P* = 0.022) in the stroma and decreased M1/M2 ratio (*P* = 0.016) in the tumor of CRS1 non-responders (**Figures 2H, J, K**). Moreover, compared with the non-responders, the responders displayed a larger proportion of tumors showing increase in the infiltration of CD20^+^ cells (*P* = 0.046) and in the M1/M2 ratio (*P* = 0.038) as well as fewer tumors showing increase in the infiltration of CD56^bright^ cells (*P* = 0.041) (**Figures 2L−O**). We also tried to examine the association between pre-NACT TIME and response to NACT and failed to find TIME markers predictive for response to NACT. With regard to the correlation between TIME post-NACT and response to NACT, CA125 responders displayed a lower density of PD-L1^+^CD68^+^ macrophages in the stroma than CA125 non-responders. Compared with CRS1 non-responders, CRS2 responders displayed a significant lower degree of infiltration of M1 macrophages (tumor, *P* = 0.006) and M2 macrophages (tumor, *P* = 0.0497) in the tumor (**Supplementary Tables S3–S6**).

### Association of TIME with prognosis

**Figure 3:**
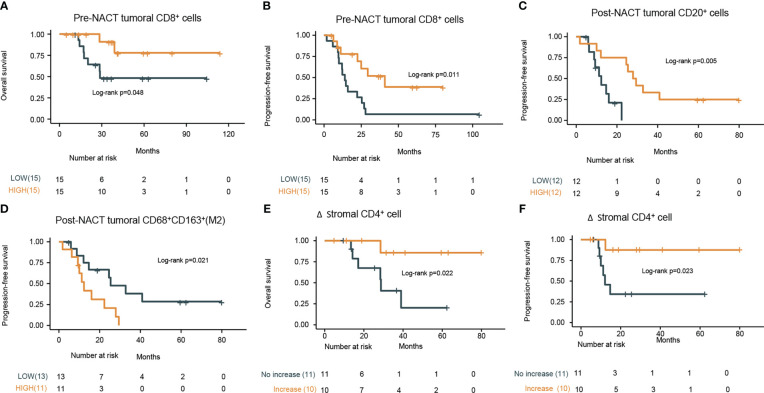
Prognostic values of pre-NACT and post-NACT immune cell density. Kaplan–Meier curves of the **(A)** overall survival (OS) and **(B)** progression-free survival (PFS) stratified by infiltration degree of pre-NACT tumoral CD8^+^ cells (low or high based on the median value) in the tumor specimens. **(C)** Kaplan–Meier curves of PFS stratified by the infiltration degree of tumoral CD20^+^ cells post-NACT (low or high based on the median value) in the tumor specimens. **(D)** Kaplan–Meier curves of PFS stratified by the infiltration degree of tumoral CD68^+^CD163^+^ (M2 macrophages) cells post-NACT (low or high based on the median value) in the tumor specimens. **(E)** Kaplan–Meier curves of the OS stratified by the increase in the density of stromal CD4^+^ cells (increase or no increase) in the tumor specimens. **(F)** Kaplan–Meier curves of the progression-free survival (PFS) stratified by the increase in the density of stromal CD4^+^ cells (increase or no increase) in the tumor specimens. P values refers to log rank tests between the indicated groups.

We also attempted to explore the predictive factors for survival. Univariate cox regression analysis of clinical characteristics revealed R0 resection and platinum sensitivity favorable for predicting better OS and PFS (**Table 2**). Subsequently, we examined the predictive value of pre-NACT TIME for survival. Using median value as a cut-off, high infiltration of CD8^+^ cells in the pre-NACT tumor was positively associated with prolonged OS (CD8^high^ vs. CD8^low^, NR vs. 28.7 months, HR, 0.23, 95% CI, 0.05-1.1, log-rank *P* = 0.048) and PFS (CD8^high^ vs. CD8^low^, 40.9 vs. 14.0 months, HR, 0.31, 95% CI, 0.12-0.79, log-rank *P* = 0.011) (**Figures 3A, B**; **Supplementary Table S7**).

**Table 2:** Univariate cox regression analysis of clinical characteristics in all available samples.

	PFS			OS		
Characteristic	HR	95%CI	p-value	HR	95%CI	p-value
Age						
≤50	Ref			Ref		
>50	2.82	0.83, 9.59	0.096	3.15	0.41, 24.5	0.272
ECOG						
I-II	Ref			Ref		
III	0.59	0.23, 1.50	0.266	0.49	0.15, 1.65	0.249
Lymph node metastasis						
No	Ref			Ref		
Yes	1.06	0.46, 2.44	0.889	1.52	0.48, 4.81	0.475
CA-125 1^st^ cycle response						
No	Ref			Ref		
Yes	0.53	0.22, 1.27	0.153	0.36	0.11, 1.20	0.097
CRS						
1	Ref			Ref		
2-3	0.91	0.33, 2.52	0.862	1.22	0.22, 6.69	0.815
R0						
No	Ref			Ref		
Yes	0.42	0.18, 0.97	0.042	0.27	0.08, 0.94	0.039
Platinum sensitivity						
Platinum resistant	Ref			Ref		
Platinum sensitive	0.09	0.03, 0.30	<0.001	0.21	0.06, 0.75	0.016

HR, hazard ratio; CI, confidence interval, Ref, reference.

When considering the predictive value of post-NAT TIME for survival, high density of CD20^+^ B cells (CD20^high^ vs. CD20^low^, mPFS, 28.65 vs. 12.30 months; HR, 0.17; 95% CI, 0.04-0.67; log-rank *P* = 0.005) (**Figure 3C**), more abundant TLS (TLS-negative vs. TLS-positive, mPFS, NR vs. 14.7 months; HR, 0.15; 95% CI, 0.02-1.1; log-rank *P* = 0.034) (Data not shown), and lower density of CD68^+^CD163^+^ M2 macrophages (M2 macrophages^high^ vs. M2 macrophages^low^, mPFS, 12.30 vs. 25.40 months; HR, 3.2; 95% CI, 1.14-9.01; log-rank *P* = 0.021) in the post-NACT tumor were found to predict better PFS (**Figure 3D**; **Supplementary Table S8**).

Considering the potential association between TIME change and PFS or OS, we stratified patients into infiltration ^increase^ and infiltration ^no increase^ subgroups according to infiltration changes of specific immune cell subsets during NACT. Compared with patients with no increase in the density of CD4^+^ cells in the stromal, patients with increase in this cell subset had significantly prolonged PFS (log-rank *P* = 0.022) and OS (log-rank *P* = 0.023) (**Figures 3E, F**; **Supplementary Table S9**). In the multivariate analysis, among R0 resection, platinum-sensitivity, tumoral CD8^+^ pre-NACT, tumoral CD20^+^ post-NACT, tumoral M2 macrophages post-NACT, and the increase of stromal CD4^+^ upon NACT, only platinum-sensitivity was independently associated with PFS (HR, 9.83; 95% CI, 1.98–48.83; *P* < 0.001), and OS (HR, 52.74; 95% CI, 1.45–1916.12; *P* = 0.031) and tumoral CD8^+^ pre-NACT (HR, 0.06; 95% CI, 0–0.91; *P* = 0.042) were independently associated with improved OS. The difference in PFS between pre-NACT CD8^high^ and pre-NACT CD8^low^ patients was marginally significant (*P* = 0.054) (**Table 3**).

**Table 3:** Multivariate cox regression analysis of potential risk factors in pre- and post-NACT paired samples.

	PFS			OS		
Characteristic	N	Event N	p-value	N	Event N	p-value
R0						
No	10	7		10	4	
Yes	11	8	0.460	11	3	0.622
Platinum sensitivity						
Platinum sensitive	4	4		4	2	
Platinum resistant	17	11	<0.001	17	5	0.031
Pre-NACT tumoral CD8+						
Low	10	10		10	5	
High	11	5	0.054	11	2	0.042
Δ Stromal CD4+						
No increase	11	9		11	6	
Increase	10	6	0.217	10	1	0.293
Post-NACT Tumoral CD20+						
Low	12	9				
High	9	6	0.081			
Post-NACT tumoral CD68^+^CD163^+^(M2)						
Low	11	6				
High	10	9	0.130			

## Discussion

In the current study, we reported that the infiltration of immune cells in the tumor lesions pre-NACT and post-NACT was associated with response to NACT and long-term survival benefits. With mIF, we analyzed multiple immune cell subsets closely associated with cancer dissemination in a cohort of patients with paired pre-NACT and post-NACT specimens. We show that NACT altered the balance of immune cell subsets of T cells, B cells, and macrophages, showing significantly elevated density of CD8^+^ T cell, CD20^+^ B cells, and PD-L1^+^CD68^+^ macrophages. The biopsies pre-NACT from patients with good response to NACT had significantly increased infiltration of CD8^+^ T cells and CD20^+^ B cells compared with tumor specimens post-NACT. Infiltrates of CD8^+^ T cells pre-NACT, CD20^+^ B cells and CD163^+^ macrophages post-NACT, and marked change in CD4^+^ T cells during NACT are associated with prolonged survival.

Previous studies have shown the effect of chemotherapeutic regimens on tumor immune microenvironment, including an increase in infiltration of CD8^+^ T cells, CD3^+^ T cells, CD20^+^ B cells, and PD-1^+^ cells (13–15, 19, 20). Regulation of tumor-promoting immune suppressors and immune effectors of the immune system might result in the enhanced antitumor activity of immunotherapies. It is, therefore, encouraging that NACT had significantly increased the infiltration of TILs in our and previous studies. Albeit no difference in the density of immune cell subsets positive for markers in the pretreatment specimens between responders and non-responders, responders displayed significantly increased infiltration of CD8^+^ T cells in the tumor, CD20^+^ T cells, PD-1^+^ cells, PD-L1^+^ cells, and PD-L1^+^CD68^+^ macrophages in the stroma; CD4^+^Foxp3^+^ Treg cell and PD-L1^+^CD68^+^ macrophage density in the stroma significantly increased in non-responders. Consistently, Bohm S et al. reported that density of Foxp3^+^ cells in biopsies from good responders significantly declined after NACT (14). Interestingly, the fact that infiltrates of PD-L1^+^CD68^+^ macrophage density increased in both responders and non-responders suggested a rationale for immunotherapy of immune checkpoint blockade after NACT for both patients responding and not-responding to NACT. Taken with the finding of a marked increase in infiltration of immune effectors in the biopsies from responders, it is rationale to speculate that TIME changes are associated with response to NACT. Of note, although PD-L1^+^ macrophages significantly elevated after NACT in both responders and non-responders, the observation that the infiltration of PD-L1^+^ macrophages in the post-NACT biopsies of responders was significantly lower than in non-responders further implies that patients resistant to NACT might be more responsive to PD-1 blockade.

We attempted to examine immune cell signatures predicting long-term survival under the following conditions. Pretreatment, first, the density of CD8^+^ T cells was positively associated with longer PFS and OS, consistent with previous reports (10, 12). Other immune cell subset markers in pretreatment biopsies were not predictive for PFS and OS in our observation. Second, post-NACT CD20^+^ and CD163^+^ macrophages (M2) were associated with prolonged and shortened PFS, respectively. There is evidence that a high M1:M2 ratio post-NACT is predictive of improved PFS and OS (13, 21), which supports our findings. The correlation of post-NACT CD20^+^ B cell with survival is a novel finding discovered herein. Third, with regard to the association between TIME change and survival, we revealed for the first time that the increase in the density of CD4^+^ T cells was predictive for longer PFS and OS. In the multivariate analysis, among R0 resection, platinum sensitivity, and the above immune cell subset markers predicting survival in the univariate analysis, only platinum-sensitive and tumoral CD8^+high^ pre-NACT were independently associated with improved OS.

This study was limited by its retrospective design and small sample size. Nevertheless, the mIF workflow employed in this study allowed the characterization of multiple biomarkers closely correlating to immune activators and suppressors, rendering it feasible to monitor TIME upon NACT in a one-set test. As we focused on the TIME change at the protein level assessed by mIF, further studies should integrate analysis from diverse confirmatory experimental methods, including mRNA sequencing, whole-exome sequencing, and immunoproteomics.

## Conclusions

This study examined the dynamics of tumor-infiltrating immune cells using the matched pre-NACT and post-NACT tissue biopsies and compared these to the response to NACT and long-term survival. Evaluation of dynamics in the TIME could help reflect NACT efficacy, predict survival benefit, and perhaps most importantly, guide rational immunotherapy after NACT rather than in relapsed disease.

## Data availability statement

The original contributions presented in the study are included in the article/**Supplementary Material**. Further inquiries can be directed to the corresponding authors.

## Ethics statement

The studies involving human participants were reviewed and approved by The ethics committee of Beijing Chao-Yang Hospital. The patients/participants provided their written informed consent to participate in this study. Written informed consent was obtained from the individual(s) for the publication of any potentially identifiable images or data included in this article.

## Author contributions

GC: Acquisition of data, writing-review and editing. DH: Methodology, writing-review and editing. JL: Data curation, formal analysis, writing-review and editing. XZ: Data curation, formal analysis, writing-review and editing. ZZ: Data curation, formal analysis, writing-review and editing. BZ: Data curation, formal analysis, writing-review and editing. TB: Writing–original draft, writing–review and editing. LC: Conceptualization, resources, supervision, writing–review and editing. SC: Conceptualization, resources, supervision, writing–review and editing. SW: Resources, data curation, writing-review and editing. LZ: Conceptualization, resources, supervision, methodology, project administration, writing–review and editing. All authors contributed to the article and approved the submitted version.
