# Supplementary material for: Tumor immune microenvironment changes are associated with response to neoadjuvant chemotherapy and long-term survival benefits in advanced epithelial ovarian cancer: A pilot study
Source: Front Immunol. 2023 Mar 13;14:1022942. doi: 10.3389/fimmu.2023.1022942 (PMC10040680; doi:10.3389/fimmu.2023.1022942)
Supplement: Supplementary file 2 [file DataSheet_2.docx]

**
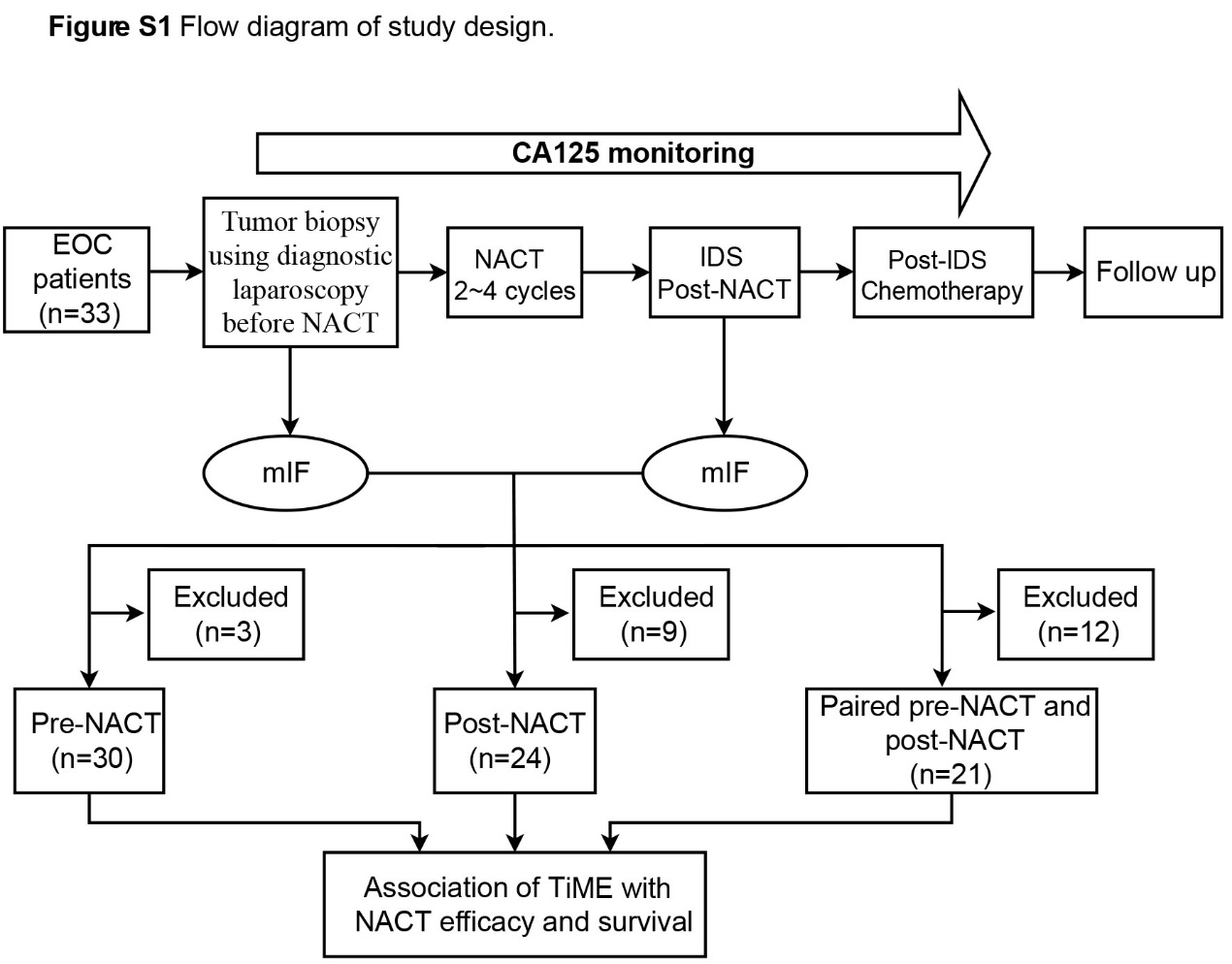
**

**Supplementary Figure 1.** Flow diagram of study design.

A total of 33 patients with paired tissue specimens collected at diagnosis and surgery were included in the study. Tissue specimens that failed to pass quality control of multiplex immunofluorescence were excluded from the subsequent analysis. OC, epithelial ovarian cancer; NACT, Neoadjuvant chemotherapy; TIME, Tumor immune microenvironment; IDS, Interval debulking surgery; mIF, multiplex immunofluorescence

**
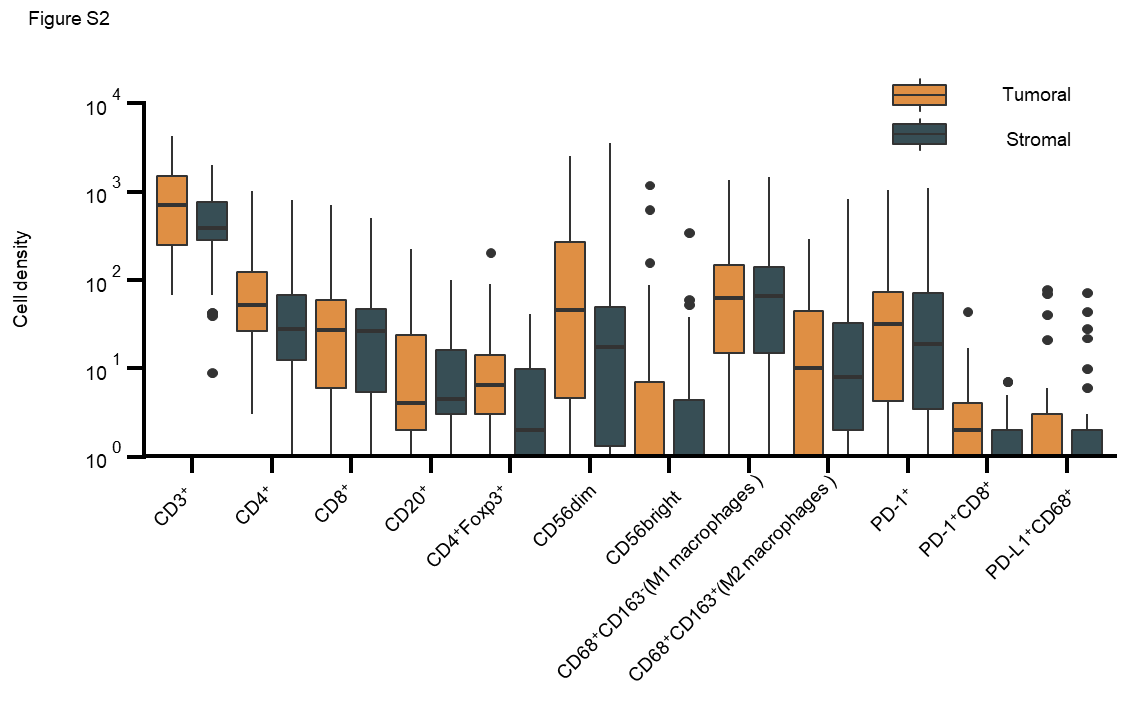
**

**Supplementary Figure 2.** Immune cell subsets in the treatment-naïve tumors from patients with advanced epithelial ovarian cancer (n=30).


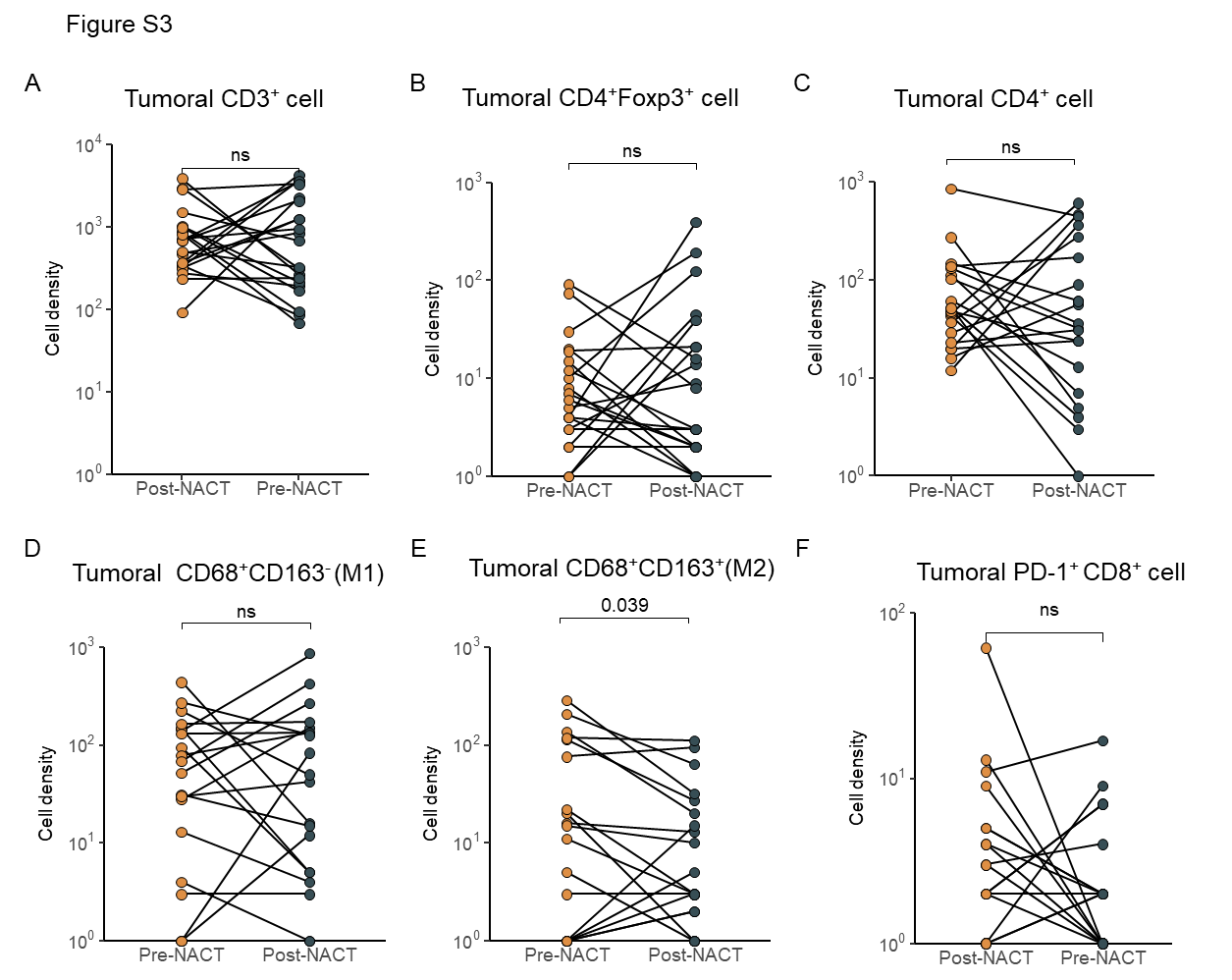


**Supplementary Figure 3.** Comparison of density of (A) tumoral CD3^+^ cell;(B) tumoral CD4^+^Foxp3^+^cell; (C) tumoral CD4^+^ cell; (D) tumoral CD68^+^CD163^-^(M1 macrophages); (E) tumoral CD68^+^CD163^+^(M2 macrophages); (F) tumoral PD-1^+^CD8^+^ cell between pre- and post-NACT tumor samples. P value, Wilcoxon matched-pairs signed-rank test.
