# Supplementary figures and images for: Tumor immune microenvironment changes are associated with response to neoadjuvant chemotherapy and long-term survival benefits in advanced epithelial ovarian cancer: A pilot study
Source: Front Immunol. 2023 Mar 13;14:1022942. doi: 10.3389/fimmu.2023.1022942 (PMC10040680; doi:10.3389/fimmu.2023.1022942)

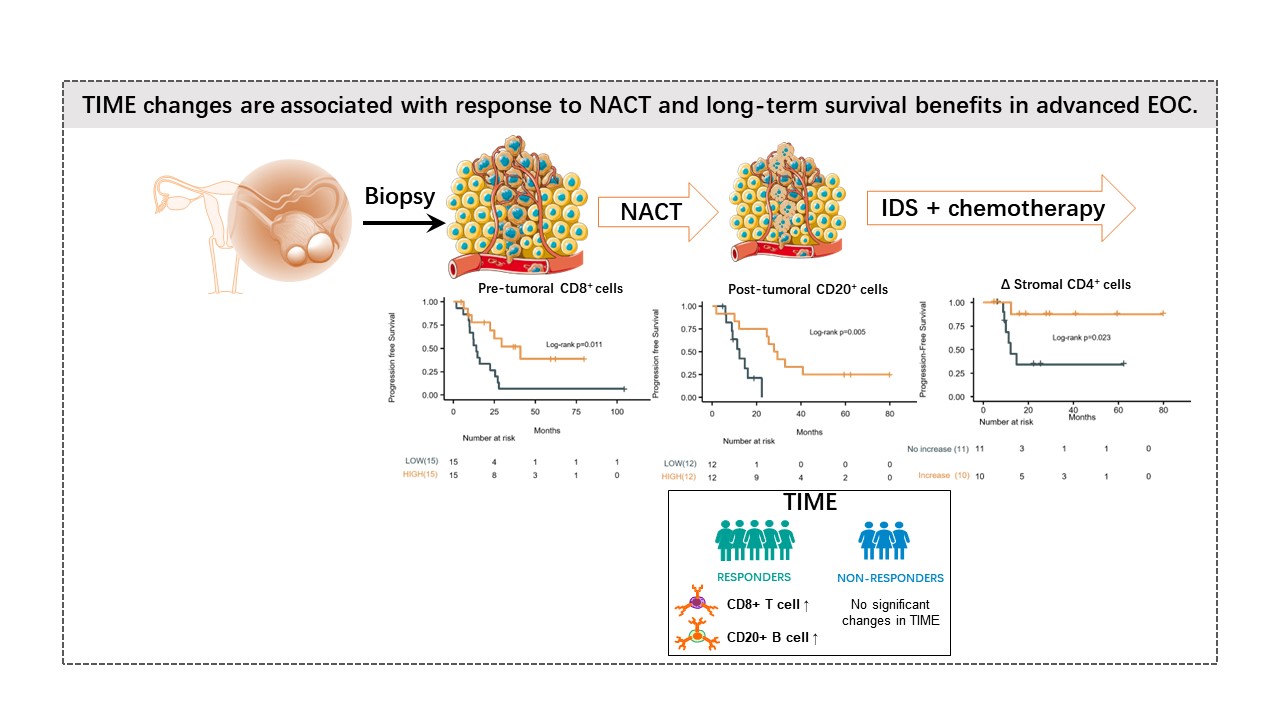

Supplement: Supplementary file 3 [file Image_1.jpeg]
